# Successful Amputation at the Hip-Joint

**Published:** 1827-09

**Authors:** Valentine Mott

**Affiliations:** Professor of Surgery at Rutgers Medical College, New York.


### AMPUTATION AT THE HIP-JOINT.

*Successful Amputation at the Hip-Joint.* By VALENTINE MOTT, M.D. Professor of Surgery at Rutgers Medical College, New York. (Condensed from the *Philadelphia Journal.*)

IT is now generally understood that surgical operations are not to be performed until all other curative measures have proved unavailing, or the life of the individual cannot be saved, unless some part be sacrificed for the preservation of the whole. We have, nevertheless, reason to rejoice that, under exceedingly unfavourable circumstances, these dreaded resources of our art afford a rational prospect of success, frequently enabling us to arrest or remove morbid affections, otherwise beyond the reach of cure, and to prolong valuable lives in a state of comparative ease. Were we disposed to enter upon such an inquiry, it might be advantageous to determine how far the outcry against surgical operations (doubtless just in numerous instances,) has proved detrimental to the interests of humanity, by causing the knife to be withheld in many cases where an intrepid and skilful employment of it would have been followed by the restoration of health, and the avoidance of the excruciating sufferings so often endured for a long time previous to the death of such patients. Without discussing this topic, however, we may be permitted to state our belief that a great number of persons are annually committed to the grave, because proper surgical measures are not enforced, and that these are as often withheld from timidity, prejudice, or ignorance, as from any valid objection.

Amputation at the hip-joint is an operation but seldom required, and always attended with great peril, both to the life of the patient and the reputation of the surgeon; but neither of these circumstances are sufficient to justify any one in asserting that this operation ought not to be performed, or that it may not in a majority of cases prove successful, if it be not too long deferred. The following case may prove serviceable to the profession, by showing that the operation may be advantageously attempted in a patient who would otherwise have speedily sunk under his disease. It is moreover interesting from the circumstance of its being the first amputation at the hip-joint successfully performed in this country, as far as our present information extends.

George Byles, a healthy boy, ten years old, broke his thigh about two-thirds of its length from the hip-joint; two days after, splints and bandages were firmly (and injudiciously) applied, which produced great distress, and were removed at the instigation of the boy. Physick's modification of Desault's splint was prepared by the physician then called in, who pointed out to the father, previous to its application, a projecting point on the outside of the thigh, which was the extremity of the superior fragment, which, by the improper pressure, was nearly forced through the integuments. The bone being properly coaptated, the long splint was then applied.

About three weeks subsequent to this period, another physician was called in, who recommended the employment of the inclined plane, which was adopted, the boards forming it having pegs at the side. The boy stated that, during his confinement to this inclined plane for several weeks, he had, in tossing restlessly about, injured the thigh on the inside just above the condyle, which produced a sinous opening leading to the fractured bone. It is most probable, however, that the sinus was formed and pointing, when it struck against the peg and opened.

He was brought into the city of New York on the 7th of September, 1824, at which time we first saw him. His countenance was expressive of much anguish, with a white tongue and feeble pulse; his right limb was much enlarged on the outside, resembling a case of spina ventosa. To the touch it was hard and irregular, was exceedingly tender, and when pressed gave excruciating pain. The swelling extended to the great trochanter, gradually diminishing towards the top of the thigh. Opposite to the greatest enlargement was a sinus, discharging a thin sanious fluid, leading to the middle of the thigh-bone, which was perfectly carious. During two weeks succeeding his arrival in the city, medicines were administered with a view of allaying irritation, and imparting tone to the system, but hectic and night-sweats, notwithstanding, supervened. As ulceration began to occur by the side of the tibia, and all the symptoms became worse, it was resolved to amputate at the hip-joint, as the only chance of saving the life of the patient.

On the 7th of October, 1824, the patient, after having passed a comfortable night, was placed upon the table in order to be operated on. An incision was made over the femoral artery, as it emerges from under the femoral arch, and the vessel secured by ligature. While feeling on the outside of the artery for the lesser trochanter, the pulsation of a vessel, apparently but little smaller than the femoral artery, immediately below the ligature, convinced us that in this case the profunda femoris was given off above the femoral arch, as we occasionally find it. This vessel was taken up.

Lisfranc's knife was then introduced between the artery and bone, and carried through close by the neck of the femur towards the tuber ischii, thus forming the inner flap. The external flap was formed by cutting from without inwards. The hemorrhage from the veins and small arteries was considerable when the incisions were made, and numerous vessels were taken up: but comparatively little blood was lost during the operation, and the patient was put to bed shortly after it was completed. After the inner flap was cut, some of the surgical attendants examined the lesser trochanter, pronounced that the head of the bone was *not diseased*. In order to satisfy the doubts expressed, the bone was sawed through the lesser trochanter, when it was found to be of the consistence of cheese, being denuded of periosteum on the outerside up towards the joint, and requiring to be removed, which was afterwards done, as originally contemplated.

It is scarcely necessary for us to enter into the detail of symptoms and treatment subsequent to the operation, as nothing occurred worthy of note, except various degrees of irritation of the stomach and whole system, previous to the coming away of the ligatures. The treatment consisted in regulating the diet, and administering anodyne and tonic medicines, according to circumstances.

On the 15th of October, eight days from the operation, two-thirds of the stump was healed by the first intention. Between the 17th and 31st of October, all the ligatures, seventeen in number, were removed; and by the 20th of November the whole stump was effectually healed, and the boy had become fat and lusty.

There can be no doubt but that this limb might have been saved without difficulty, had the proper treatment been instituted when the accident occurred. When it came under our charge, nothing short of the operation above related could have saved this boy's life.

